# E-learning recommender system dataset

**DOI:** 10.1016/j.dib.2023.108942

**Published:** 2023-02-01

**Authors:** Mounir Hafsa, Pamela Wattebled, Julie Jacques, Laetitia Jourdan

**Affiliations:** a Mandarine Academy, Lille, France; b University of Lille, CNRS, Centrale Lille, UMR 9189 – CRIStAL, Lille, France

**Keywords:** Recommender systems, E-Learning, MOOC, Implicit ratings, Explicit ratings, Collaborative filtering

## Abstract

Mandarine Academy is an Ed-Tech company that specializes in innovative corporate training techniques such as personalized Massive Open Online Courses (MOOCs), web conferences, etc. With more than 550K users spread across 100 active e-learning platforms.

The company creates online pedagogical content (videos, quizzes, documents, etc.) on daily basis to support the digitization of work environments and to keep up with current trends. Mandarine Academy provided us with access to Mooc.office365-training.com. A publicly available MOOC in both French and English versions to conduct research on recommender systems in online learning environments.

Mandarine Academy collects user feedback using two types of ratings: Explicit (Like Button, Social share, Bookmarks), and Implicit (Watch Time, Page View). Unfortunately, explicit ratings are underutilized. Most users avoid the burden of stating their preferences explicitly. To address this, we shift our attention to implicit interactions, which generate more data that can be significant in some cases. Implicit Ratings are what constitute Mandarine Academy Recommender System (MARS) Dataset.

We believe that the degree of viewing has an impact on the overall impression, for this reason, we applied changes to the implicit data and made a part of it similar to the explicit rating format found in other known datasets (e.g., Movielens).

This paper presents two real-world dataset variations that consist of 89,000 explicit ratings and 276,000 implicit ratings. Data was collected starting early 2016 until late 2021. Chosen users had rated at least one item. To protect their privacy, sensitive information has been removed. To the best of our knowledge, this is the first publicly available real-world dataset of E-Learning recommendations in both French and English with mixed ratings (implicit and explicit), allowing the research community to focus on pre-and post-COVID-19 behavior in online learning.

**Specifications Table**

**Table:** 

Subject	Information Systems and Applied Machine Learning
Specific subject area	Recommender Systems and Information Retrieval
Type of data	Tables (.csv)
How the data were acquired	Data were acquired by ingesting user tracking data from a relational database using Python scripts.
Data format	Data files are filtered and analyzed. Available in comma-separated values (*.csv) files.
Description of data collection	The data was collected through Mandarine Academy's Public MOOC: (mooc.office365-training.com). Data was exported in “comma separated values” (.csv) files from the company's database (MySQL). We focus on implicit interactions, which collect users’ behavior in two formats (watch time and page views). We selected events from early 2016 until late 2021. We also removed any indication of the user's identity. Users that didn't contribute to at least one event are discarded. Items that aren't used inside the platform (inactive) are not included in the dataset.
Data source location	Institution: Mandarine Academy City/Town/Region: Villeneuve D'ascq, Lille Country: France
Data accessibility	Repository name: Harvard Dataverse (E-learning Recommender System Dataset) [2] Direct URL to data: https://doi.org/10.7910/DVN/BMY3UD
Related research article	Mounir Hafsa, Pamela Wattebled, Julie Jacques, and Laetitia Jourdan. 2022. Multi-objective Recommender System for Corporate MOOC. In Proceedings of the Genetic and Evolutionary Computation Conference Companion (GECCO ’22). Association for Computing Machinery, New York, NY, USA, 2314–2317. https://doi.org/10.1145/3520304.3534058

## Value of the Data

• The dataset is comprised of real-world user ratings collected from a public E-Learning platform (MOOC.office365-training.com).
• Unlike other publicly available recommendation datasets (e.g., Last.fm [1] or Movielens [6]), this dataset includes both implicit and explicit interactions for the same context. This enables researchers to apply approaches that were previously limited to a particular rating type.
• Researchers, software developers, and industry professionals can use the dataset to benchmark future and current recommendation algorithms, as well as Information Retrieval (IR) and Machine Learning (ML) approaches.
• The MARS dataset enables the analysis and modeling of changes in online learning behavior before and after the COVID-19 pandemic.
• This dataset is compatible with a variety of techniques, including Machine Learning (ML) and Deep Learning (DL) models:
  1. Content-based Filtering (CBF): This recommendation technique creates a user profile based on their previous behaviors, whether explicit (e.g., likes, shared content, etc.) or implicit (e.g., page views). Furthermore, this strategy takes advantage of content attributes (numerical or textual) to find other items with comparable characteristics [4].
  2. Collaborative Filtering (CF): This technique relies heavily on ratings (implicit or explicit) rather than content descriptors. Using rating patterns, this method can locate similar persons (User-based CF) or similar items (Item-based CF). Machine and Deep Learning models may also be used to predict user ratings on unseen items (Model-based) [5].
  3. Association Rules: This approach primarily uses implicit or explicit ratings to find common patterns, correlations, and relationships between content [3].
  4. Popularity Models: The most basic type of recommendation, this approach employs implicit or explicit user ratings to propose the most visited, watched, or clicked items.
  5. Hybrid recommendation techniques: These are strategies that combine two or more of the methods listed above.
  6. Natural Language Processing (NLP): This method automatically manipulates natural language like textual information to make predictions. Using NLP methods with our dataset to find similarities between items is possible through available textual features such as video subtitles, titles, tags, and descriptions in both French/English.
  7. Outlier Removal and Feature Engineering Approaches: Since our data is made up of user events, spotting outliers is possible in this scenario because data is supplied without filtering out extreme users (users with an abnormal amount of seen items). Similarly, item descriptors are supplied in a variety of formats (numerical, textual, categorical), indicating the possible application of feature engineering approaches.

## 1 Data Description

Recommender systems are often used to provide content suggestions to consumers based on their preferences.

This description identifies three primary entities: users (users.csv), items (items.csv), and ratings

(explicit/implicit ratings.csv). Each entity and its associated dataset file are described in depth below. This comprises data type, statistical information, and a description of the attributes. Since the MARS dataset is based on the Mooc-office365-training platform data, we provide a statistical summary of both the French and English versions of the platform.

Starting with the user entity, which can be found in the MARS dataset in the file “users.csv”. Table 1 gives a summary of the features in the file for both the French and English versions. It should be noted that only the user's unique identity (anonymized) and job categories are utilized to secure users’ personal information and reinforce privacy measures. The“ Job” feature displays the user's job title. However, most users do not indicate this because it is not required, resulting in a substantial missing count. From the count column, we can see that the French version of the dataset has more users compared to the English version. We next examine the items entity, which can be found in the MARS dataset in the file “items.csv”. Table 2 gives a summary of the features in the file for both the French and English versions. Resources are delivered in a video format with the possibility to select subtitles among 11 languages. Note that subtitles are found in the description column. Resources can have a specific type, either tutorial, use case, or webcast. The difference is the average video length. For tutorials and use cases, the average video length is 2-3 minutes while webcasts have an average duration of 30 minutes. Each resource can be associated with specific tags, found in“ job“, “software”, and “theme”. However, we can see that both the level and job tags are mostly missing from the data. Similar to the “users” file, the items in the French version (1451) of the platform have more content compared to the English version (1167).

**Table 1:** Overview of user features (users.csv).

Feature	Description	Type	Count		Missing	
			FR	EN	FR	EN
User ID	Unique identifier	Int	121345	9902	0%	0%
Job	Job category	Category	9 057	1 409	92.53%	85.7

**Table 2:** Overview of item features (items.csv).

Feature	Description	Type	Count		Missing	
			FR	EN	FR	EN
Item ID	Unique identifier	Int	1451	1167	0%	0%
Language	Job category	Category	1451	1167	0%	0%
Title	Content title	Text	1451	1167	0%	0%
Views	Number of views	Int	1392	1119	4%	4%
Description	Content description	Text	1221	1009	15.85%	13.53%
Creation Date	Content upload date	Date	1451	1167	0%	0%
Duration	Duration in seconds	Int	1451	1167	0%	0%
Type	Tutorial, Use Case, or Webcast	Category	1451	1167	0%	0%
Level	Beginner, Intermediate, Advanced or Undefined	Category	349	475	75.94%	59.29%
Job	Related professions	Category	326	1167	77.53%	74.46%
Software	Related software	Category	1349	1167	7%	5.31%
Theme	Related theme	Category	1269	1167	12.54%	25.44%

Moving on to explore the dataset's implicit and explicit ratings. Both ratings are collected implicitly in reality, but in the context of the e-learning recommender system, watch time (implicit) is considered explicit, since users voluntarily view the training video to confirm their learning objectives. The first file is “explicit ratings.csv“ which details the amount of time each user spent watching a video. The characteristics are summarized in Table 3.

**Table 3:** Overview of explicit ratings (Watch time) (explicit ratings.csv).

Feature	Description	Type	Unique Count		Total Count	
			FR	EN	FR	EN
Item ID	Unique identifier	Int	1350 (93%)	776 (66.49%)	85339	3659
User ID	Unique identifier	Int	9789 (8%)	822 (8.3%)		
Watch %	Watch percentage of video	Float	-	-		
Rating	Watch percentage scaled on 1-10	Category				
Creation Date	Event date	Date				

Please be aware that the “Unique Count” column displays the number of unique items/users, as well as their percentage of the overall number of users and items on the platform. The “Watch %” column displays a value ranging from 0 (the video didn't start) to 100 (the video finished). The “Rating” column, on the other hand, converts the “Watch %” column to a (1-10) scale to facilitate rating comparison. Overall, both columns are seen as a more straightforward approach to determining the user's degree of interest. Duplicate observations of (user, item) pairs are found, however, the “creation date” distinguishes these observations.

**Fig. 1:**
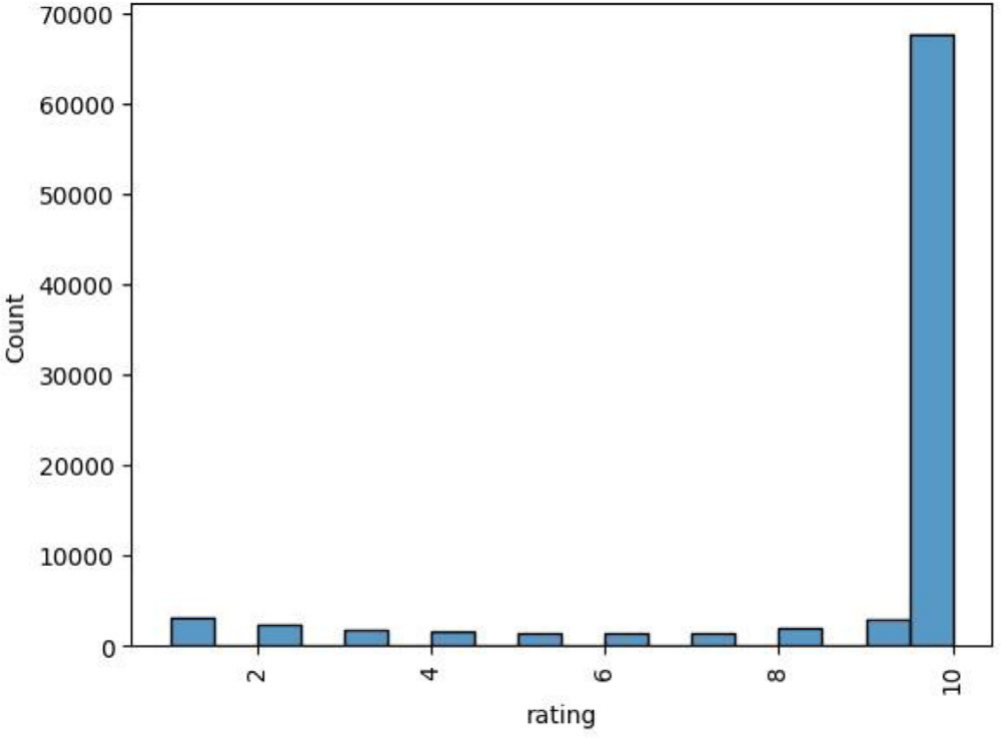
“Rating” column values distribution (French version) (explicit ratings.csv).

**Fig. 2:**
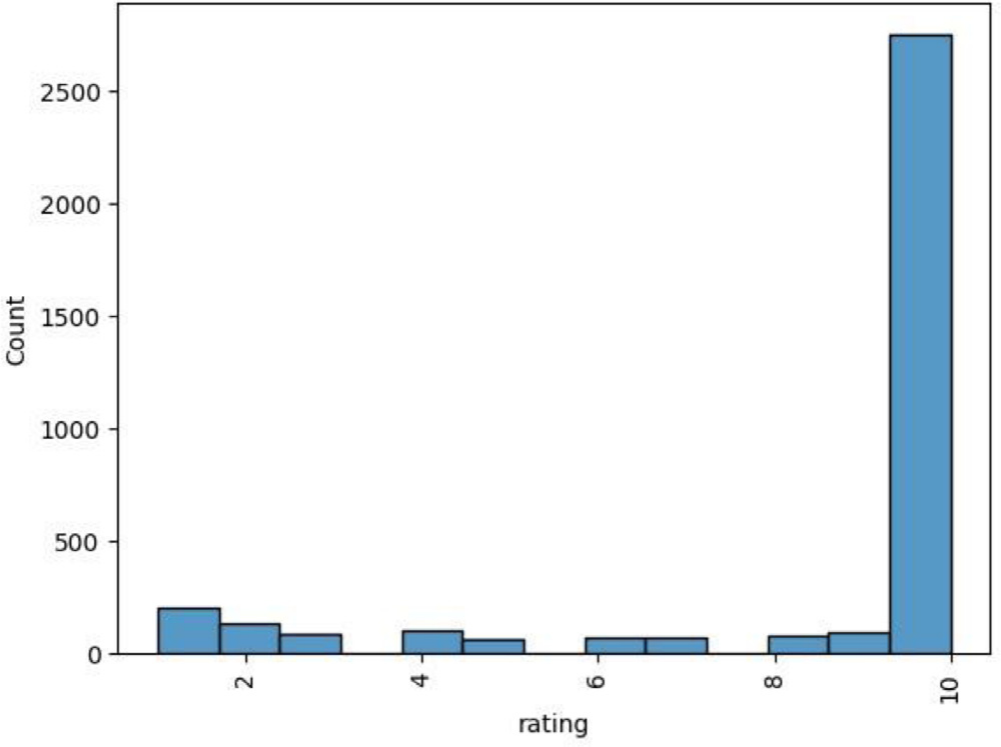
“Rating” column values distribution (English version) (explicit ratings.csv).

In Table 4, we define the logic behind associating the “Watch %” values to the “Rating” column. Finally, Figs. 1 and 2 shows how the “Rating” column is distributed in both the French and English versions (explicit ratings).

**Table 4:** Observations relative to the “Rating” and “Watch %” columns (explicit ratings.csv).

Watch percentage (From % -To %)	Proposed rating	Proposed observation
0% – 10%	1	No interest
11% - 20%	2	
21% - 30%	3	Small interest
31% - 40%	4	
41% - 50%	5	Medium interest
51% - 60%	6	
61% - 70%	7	High interest
71% - 80%	8	
81% - 90%	9	Finished
91% - 100%	10

Finally, the implicit ratings file “implicit ratings.csv” displays platform users’ browsing history for both the French and English versions. This dataset file has more observations than the “explicit ratings.csv” file, as well as higher item/user coverage. The characteristics are summarized in Table 5. This file essentially maintains track of which user viewed which item page. Duplicate observations of (user, item) pairs are found, however, the “creation date” distinguishes these observations.

**Table 5:** Overview of implicit ratings (Item page view) (implicit ratings.csv).

Feature	Description	Type	Unique Count		Total Count	
			FR	EN	FR	EN
**Item ID**	Unique identifier	Int	1377 (94.90%)	957 (82%)	253827	21908
**User ID**	Unique identifier	Int	18519 (15.26%)	3007 (30.36%)		
**Creation Date**	Event date	Date	-	-		

## 2 Experimental Design, Materials, and Methods

This section contains a detailed explanation of the experimental design used to generate the MARS dataset.

**Fig. 3:**
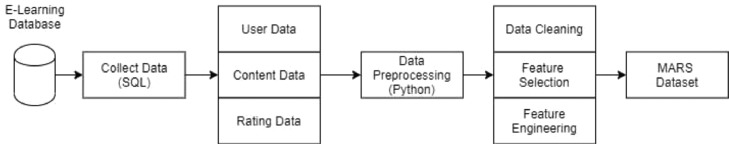
Overview of the MARS dataset experimental design.

Fig. 3 depicts the many steps required in gathering, cleaning, and verifying the dataset.

Beginning with data collection, which involves importing log data from the database (MySQL). The e-learning platform stores ratings (implicit and explicit) as well as catalog information (users and items) in a relational database. After gathering the necessary data, we go on to the next phase, which is data preparation in Python. Cleaning, selecting, and engineering features are all part of this step.

Starting with user data, the initial step was to delete inactive user accounts, then remove privacy-sensitive features before anonymizing the person's unique identity. This procedure complies with the European Union's General Data Protection Regulation (GDPR).

The second stage is to clean up the content data. We begin this procedure by deleting inactive items (those that are not publicly viewable) and ones that lack critical features (e.g., title, duration, and creation date). The second stage is replacing empty columns (e.g., “Job,” “Level,” “Software,” and “Theme”) with nulls rather than empty text. This is required to determine the proportion of missing data per feature. The final stage is to clean textual (“Description” column) data using Natural Language Processing (NLP) methods. This includes the removal of special characters, HTML elements, and stop-words. Essentially, only numbers and letters (alphanumeric) are accepted in the “Description” column.

Working with ratings is the third and last phase. Starting with implicit ratings (page views), the initial task was to remove observations with no User ID (visitors), maintaining only observations with valid (Item ID and User ID) pairs. Moving on to explicit ratings (e.g., watch time), the first objective was to only keep observations with active and existing User and Item IDs. The second step includes adjusting collected “Watch %” values that are more than the maximum set limit (100%). This step changes any “Watch %” value greater than 100 to the maximum set limit. The final phase, and the most significant contribution to this dataset, was the addition of a new scale to assess user interest. The “Rating” column is constructed by consolidating the “Watch %” values into a simpler 1-10 range.

The above processes are performed for both platform catalogs (French and English), with the final files exported as comma-separated values (.csv) files.

## Ethics Statements

Data can be used for multiple purposes within the same research domain. The dataset contains no sensitive information. This work does not entail gathering information from social media platforms. The only data source used in this work is the company's relational database, which does not include any user-related social media information. Because the data used in this study has already been anonymized, further anonymization before sharing is not required. Mandarine Academy owns this dataset. The dataset is not linked to any third-party apps or platforms.

## CRediT authorship contribution statement

**Mounir Hafsa:** Investigation, Writing – original draft, Software. **Pamela Wattebled:** Supervision, Project administration, Writing – review & editing. **Julie Jacques:** Supervision, Writing – review & editing. **Laetitia Jourdan:** Supervision, Project administration, Writing – review & editing.

## Declaration of Competing Interest

The authors declare that they have no known competing financial interests or personal relationships that could have appeared to influence the work reported in this paper.

## Data Availability

E-learning Recommender System Dataset (Original data) (Dataverse).
